# Diffractionless transmission of optical beams through a four-level atomic system affected by a plasmonic nanostructure

**DOI:** 10.1038/s41598-024-67019-4

**Published:** 2024-07-14

**Authors:** Parvin Lotfi, Mostafa Sahrai, Vahid Siahpoush, Azar Vafafard

**Affiliations:** Faculty of Physics, University of Tabriz, Tabriz, Iran

**Keywords:** Optics and photonics, Quantum optics

## Abstract

A nondiffracting propagation of an optical beam through a four-level double-V-type quantum system near a plasmonic nanostructure is investigated. We study the linear absorption and dispersion properties of the quantum system as it interacts with two laser fields. We discuss the effect of the control beam with a Laguerre-Gaussian (LG) profile on the focusing of the probe beam in the presence of a plasmonic nanostructure. An appropriately selected control beam excites one transition of the atomic system and generates a spatially varying refraction index modulation for a weak probe beam that couples to the other transition. We demonstrate that placing a plasmonic nanostructure at a nanometer distance from the atomic system and using a control field with the spatial structure leads to the diffraction-less propagation of the probe beam through the atomic system. Also, it is shown that the optical properties and probe beam focusing can be controlled by adjusting the distance of the plasmonic nanostructure from the atomic system. The proposed all-optical waveguide with high contrast and transmission can be used to implement applications such as image transfer through the medium and image processing.

## Introduction

It is well known that optical diffraction causes a fundamental restriction to imaging and light focusing in the required small area for optical instruments and microscopy. Diffraction spreading unpleasantly affects light propagation, which occurs both in free space and in a medium. Due to the wide range of applications in areas such as the transmission of small images, steering of light beams, and lithography, several solutions have been developed to overcome such a limitation.

It has been revealed that the diffraction of the light beam during propagation can be manipulated by passing the light beam through a medium with spatially varying absorption and dispersion. A control field with a spatial profile makes the medium inhomogeneous; therefore, the susceptibility of the medium depends on the intensity and profile of the strong control beam^1,2^. In this technique, quantum coherence effects such as electromagnetically induced transparency (EIT)^3–6^, coherent population trapping (CPT)^7–9^, or saturated absorption techniques^10^ are important in adjusting the optical properties to suppress diffraction.

The report of an experiment based on the proposed method demonstrated that spatial effects along with the generation of EIT pulses lead to diffraction-limited beam propagation in an optically thick medium^11^. Using the EIT, induced by laser fields^12,13^, light passes through the atomic system without absorption because of the quantum interference of multiple pathways. In an interesting study, a control laser field with a Gaussian profile was applied to generate a spatially varying refractive index in an EIT medium, and focusing on the weak traveling laser field was subsequently observed^14^. This beam-focusing technique which is known as electromagnetically induced focusing (EIF) has also been studied in a cold atomic system^15^. To achieve a better performance, Verma and his co-worker have shown that a microwave field driving the hyperfine levels of the $\Lambda$-system results in a strong EIF^16^.

Moreover, atomic vapors have been employed to create a lossless waveguide without diffraction. The results have shown a tunable waveguide-like feature inside the atomic medium using Gaussian Raman and LG control fields. Such a waveguide can guide arbitrary modes of a weak probe beam to several Rayleigh lengths without diffraction or absorption^17,18^. Also reported results confirm unambiguously that focusing of the probe beam is achieved due to the spatially varying refractive index, and the absorption and diffraction of the probe beam are very sensitive to the pump. So considering a suitable pump beam leads to more effective production of diffraction-limited propagation^19^.

On the other hand, surface plasmon provides innovative and distinctive methods for manipulating light due to the collective oscillations of free electrons in metallic nanostructures. Such a property results in a whole set of new behaviors and applications^20–25^. The effects of spontaneous decay and resonance fluorescence of quantum emitters placed near a plasmonic structure have attracted much attention since the 1970s^26–28^. It is worth noting that several interesting experiments have reported that the optical properties near plasmonic nanostructures behave quite differently than in free space^29–36^. An experimental study has demonstrated the fluorescence rate of a single molecule as a function of its distance to a laser-irradiated gold nanoparticle^31^. The proposed experimental arrangement has revealed that by varying the distance between the molecule and the gold nanoparticle, a continuous transition occurs from fluorescence enhancement to fluorescence quenching.

Furthermore, quantum coherence and interference effects in atoms, molecules, and semiconductor nanostructures near or within plasmonic microstructures and nanostructures have also been investigated^37,38^. One of the studies in this area that has become increasingly popular is the effect of plasmonic on quantum interference between two spontaneous emission channels of a three-level V-type atom system. Quantum interference due to surface plasmon effects has been shown to increase when the quantum system is placed near the plasmonic nanostructure^39^. Moreover, the adjustment of free-space spontaneous emission rates of the V-type atomic system in the presence of the plasmonic nanostructure has been studied^40^. In such a system, the distance between the atoms and the plasmonic nanostructure plays a key role in the spontaneous emission rate. In our recent work, we considered a double-V-type atomic system near the plasmonic nanostructure, which interacts with a standing-wave coupling light^41^. We have shown that transferring the probe energy from zero order to high order diffraction can be possible by changing the distance between the atomic system and the plasmonic nanostructure.

In this paper, we present an exclusive method to adjust the light focus. The idea is to modify the atomic decay rates by placing a plasmonic nanostructure near a four-level atomic system. The proposed model contains a double V-type atomic system that interacts with a weak probe light, and also couples to an excited state by a control light with an LG profile. We first investigate the effect of plasmonic nanostructure on the optical properties of the atomic system. Transparency and gain without inversion as well as nonzero dispersion can be achieved by placing the plasmonic nanostructure close to the atomic system. The distance between the plasmonic nanostructure and the atomic system is very advantageous in adjusting the spontaneous emission rate. Then, we investigate the propagation of a Gaussian probe field without diffraction through the proposed atomic system. Applying the LG control beam provides a spatially modulated refraction index in the atoms. Achieved results show that the spatial structure of the control field assisted with plasmonic nanostructures can be employed to transmit the probe beam over a large distance without diffraction and attenuation. The transmission of an image without much loss of resolution as a result of diffraction is an important issue. Due to the wide range of applications in areas such as the transmission of small images, steering of light beams, and lithography, several solutions have been developed to demonstrate a way for nondiffracting propagation of an optical beam. The proposed all-optical waveguide can be used to implement applications such as image transfer through the medium, image processing, and high-contrast imaging.

## Model and equations

**Figure 1:**
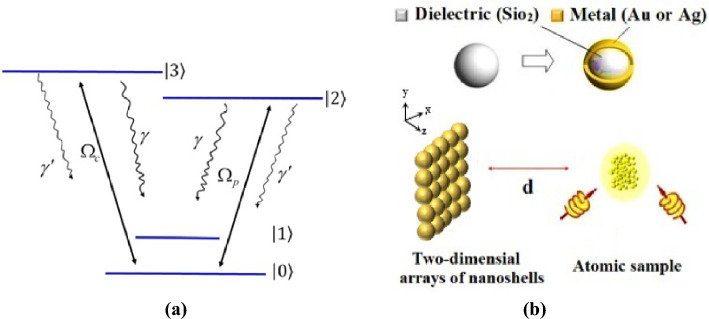
(**a**) The quantum system is a four-level double-V-type system, where the two upper levels $\left| 2 \right\rangle$ and $\left| 3 \right\rangle$ decay spontaneously to the two lower levels $\left| 0 \right\rangle$ and $\left| 1 \right\rangle$. The system interacts with a probe laser field that couples level $\left| 0 \right\rangle$ to level $\left| 2 \right\rangle$ and the control field couples level $\left| 0 \right\rangle$ to level $\left| 3 \right\rangle$, (**b**) a metal-coated dielectric nanosphere in a 2D array of such spheres near a quantum system interacting with two laser fields.

The system considered here is a double-V-type four-level atomic system with two closely lying upper levels $\left| 2 \right\rangle$ and $\left| 3 \right\rangle$ and two nearly lower levels $\left| 0 \right\rangle$ and $\left| 1 \right\rangle$ (Fig. 1a). The levels $\left| 2 \right\rangle$ and $\left| 3 \right\rangle$ characterize two Zeeman sublevels. Such a system can be generated in $D_{1}$ transitions of cold $^{87}Rb$ atoms in a vapor cell.

The dipole moment operator is taken as $\small \mathbf {\mu }=\mu ^{'}(\left| 2 \right\rangle \left\langle 0\right| \hat{\varepsilon }_{-}+\left| 3 \right\rangle \left\langle 0\right| \hat{\varepsilon }_{+})+\mu (\left| 2 \right\rangle \left\langle 1\right| \hat{\varepsilon }_{-}+\left| 3 \right\rangle \left\langle 1\right| \hat{\varepsilon }_{+})+H.c.,$ where $\hat{\varepsilon }_{ \pm } = \frac{{(e_{z} ) \pm (ie_{x} )}}{{\sqrt 2 }}$ describes the right-rotating($\hat{\varepsilon }_{+}$) and left-rotating($\hat{\varepsilon }_{-}$) unit vectors. Here, $\mu$ and $\mu ^{'}$ are taken to be real.

The double-V-type quantum system can be generated in hyperfine sublevels of D lines in $^{87}Rb$ [4,9,15]. The lower states are the two hyperfine levels $\left| 0 \right\rangle =5 S_{1/2}(F=1, m_{F}=0)$ and $\left| 1 \right\rangle =5 S_{1/2}(F=2, m_{F}=0)$. The upper states are $\left| 2 \right\rangle =5 P_{1/2}(F^{'}=2, m_{F}=-1)$ and $\left| 3 \right\rangle =5 P_{1/2}(F^{'}=2, m_{F}=1)$.

It is worth noting that according to the selection rules, transitions with $\Delta _{m}=\pm 1$ require circularly polarized light. The sign ($+$) is for right-handed and the sign ($-$) is for the left-handed circular polarization. Therefore, the probe and control beams have orthogonal polarizations to excite the corresponding atomic transitions.

The energy difference between two excited states can be tuned by a static magnetic field. For the calculation, we have used typical parameters for such an atomic system. $\Gamma _{0}=2\pi \times 5.746$ MHz is the decay rate of states $\left| 2 \right\rangle$ and $\left| 3 \right\rangle$ to state $\left| 1 \right\rangle$ in the vacuum. The dipole moment of the probe transition is considered $\mu =2.537\times 10^{-29}$ C.m. Other parameters are scaled in units of $\Gamma _{0}$.

The quantum system interacts with two circularly polarized continuous electromagnetic waves and the total electric field is given by

1 $$\begin{aligned} {\textbf{E}(t)= \hat{\varepsilon }_{+}g_p\cos (\omega _pt)+\hat{\varepsilon }_{-}g_c\cos (\omega _ct)}, \end{aligned}$$

where $g_{p}$ and $g_{c}$ are the electric-field amplitude, while $\omega _{p}$ and $\omega _{c}$ are the angular frequencies of probe and control fields, respectively. We assume that both fields have equal frequencies $\omega _p=\omega _c=\omega$.

The quantum system is placed at a distance *d* from the surface of a plasmonic nanostructure. It is assumed that both the quantum system and the plasmonic nanostructure are put in a vacuum.

The possible experimental setup consists of an external cavity tunable diode laser. The produced beam is split in two at a 50/50 beam splitter. One of these beams can pass through an acousto-optic modulator to be frequency shifted and acts as a probe field. The other beam has the role of a control beam which can be sent through a spiral phase plate to generate a donut shaped LG beam. Then, the control and probe fields are collective at a polarizing beam splitter and radiates to the atomic system. The Rb vapor cell is near the nanostructure, whose surface plasmon resonance was excited by illumination from a 532 nm (green) laser. The control beam is removed at the second polarizing beam splitter located after the cell. Such an arrangement can be prepared with the help of some interesting experimental studies^11,14^.

The plasmonic nanostructure used in this research is two-dimensional array of touching gold-coated silica nanospheres (see Fig. 1b). The proposed structure can be fabricated via self-assembly^42^ and nanopatterning-nanolithographic^43^ techniques. The dielectric constant of the gold can be obtained by a Drude model as follows^44^:

2 $$\begin{aligned} \epsilon (\omega )=1-\frac{\omega _{p}^{2}}{\omega (\omega +i b)}, \end{aligned}$$

here $\omega _{p}$ and *b* are the bulk plasma frequency and the electron collision frequency, respectively. The value of the plasma frequency for gold is $\hbar \omega _{p}=8.99 ev$. The dielectric constant of SiO2 is equal to $\epsilon =2.1$. The nano-sphere with radius $c/\omega _{p}$ and core radius $0.7c/\omega _{p}$ are located in a square lattice with a lattice constant $2c/\omega _{p}$. *c* is the velocity of light in the vacuum.

The interaction Hamiltonian of the quantum system, in the dipole and rotating-wave approximations, is given by

3 $$\begin{aligned} H= & {} \hbar (-\delta -\frac{\omega _{32}}{2})\left| 2 \right\rangle \left\langle 2\right| +\hbar (-\delta +\frac{\omega _{32}}{2})\left| 3 \right\rangle \left\langle 3\right| -(\frac{\hbar \Omega _p}{2}\left| 0 \right\rangle \left\langle 2\right| +\frac{\hbar \Omega _c}{2}\left| 0 \right\rangle \left\langle 3\right| +H.C.). \end{aligned}$$

$\delta =\omega -\tilde{\omega }$ is the detuning of the fields from the average transition energies. $\tilde{\omega } = [(\omega _{3} + \omega _{2} )/2] - \omega _{0}$ denotes the average transition energies of levels $\left| 2 \right\rangle$ and $\left| 3 \right\rangle$ from level $\left| 0 \right\rangle$. The parameters $\Omega _p$ and $\Omega _c$ describe the Rabi-frequencies of the probe and control fields, respectively. The Rabi-frequencies are defined as $\Omega _p=\mathbf {\mu }g_p/\hbar$ and $\Omega _c=\mathbf {\mu }g_c/\hbar$. Also, the energy of level $\left| n \right\rangle$ is given by $\hbar \omega _n$, $n=$0–3. We note that Eq. (3) describes only the interaction with the external laser fields. The quantum system also interacts with the vacuum fluctuation that leads to the spontaneous emission^45^. Both excited levels $\left| 2 \right\rangle$ and $\left| 3 \right\rangle$ decay spontaneously to levels $\left| 0 \right\rangle$ and $\left| 1 \right\rangle$ with decay rates $2\gamma _2^{'}$ , $2\gamma _3^{'}$, $2\gamma _2$ and $2\gamma _3$, respectively. We assume that transitions from levels $\left| 2 \right\rangle$ and $\left| 3 \right\rangle$ to level $\left| 1 \right\rangle$ lie within the surface-plasmon bands of the plasmonic nanostructure, whereas transitions from levels $\left| 2 \right\rangle$ and $\left| 3 \right\rangle$ to level $\left| 0 \right\rangle$ are spectrally distant from the surface-plasmon bands. Therefore, transitions from levels $\left| 2 \right\rangle$ and $\left| 3 \right\rangle$ to level $\left| 0 \right\rangle$ are not affected by the plasmonic nanostructure and spontaneous emission is due to the interaction of the quantum system with free-space vacuum modes. We consider the energy difference of two upper levels ($\omega _{32}=\omega _{3}-\omega _{2}$) as small as a few $\Gamma _0$ ,where $\Gamma _0$ is the decay rate of levels $\left| 2 \right\rangle$ and $\left| 3 \right\rangle$ to level $\left| 1 \right\rangle$ in the vacuum. The density matrixe quations of motion in the rotating frame are given by:

4 $$\begin{aligned} \dot{\rho _{00}}= & {} 2\gamma ^{'}(\rho _{22}+\rho _{33})-i\frac{\Omega _p}{2}(\rho _{02}-\rho _{20})-i\frac{\Omega _c}{2}(\rho _{03}-\rho _{30}),\nonumber \\ \dot{\rho _{22}}= & {} -2(\gamma +\gamma ^{'})\rho _{22}+i\frac{\Omega _p}{2}(\rho _{02}-\rho _{20})-\kappa (\rho _{23}-\rho _{32}),\nonumber \\ \dot{\rho _{33}}= & {} -2(\gamma +\gamma ^{'})\rho _{33}+i\frac{\Omega _c}{2}(\rho _{03}-\rho _{30})-\kappa (\rho _{23}-\rho _{32}),\nonumber \\ \dot{\rho _{20}}= & {} (i\delta +i\frac{\omega _{32}}{2}-\gamma -\gamma ^{'})\rho _{20}+i\frac{\Omega _p}{2}(\rho _{00}-\rho _{22})-i\frac{\Omega _c}{2}\rho _{23}-\kappa \rho _{30},\nonumber \\ \dot{\rho _{30}}= & {} (i\delta -i\frac{\omega _{32}}{2}-\gamma -\gamma ^{'})\rho _{30}+i\frac{\Omega _c}{2}(\rho _{00}-\rho _{33})-i\frac{\Omega _p}{2}\rho _{32}-\kappa \rho _{20},\nonumber \\ \dot{\rho _{23}}= & {} (i\omega _{32}-2\gamma -2\gamma ^{'})\rho _{23}+i\frac{\Omega _p}{2}\rho _{03}-i\frac{\Omega _c}{2}\rho _{02}-\kappa (\rho _{22}-\rho _{33}), \end{aligned}$$

where, $\rho _{00}+\rho _{11}+\rho _{22}+\rho _{33}=1$ and $\small \rho _{mn}=\rho ^*_{nm}.$ As already mentioned, the two upper levels $\left| 2 \right\rangle$ and $\left| 3 \right\rangle$ are considered degenerate Zeeman sublevels, so $\omega _{21}=\omega _{31}=\tilde{\omega }$. Therefore, we choose $\gamma _2=\gamma _3=\gamma$ and $\gamma _2^{'}=\gamma _3^{'}=\gamma ^{'}$^40^. The terms associated with the spontaneous emission processes are calculated by the system reserviour theory. The term $\kappa$ is the responsible for quantum interferences between the decay channels of close-lying levels due to the anisotropy of the vacuum field^46^. The values of $\gamma$ and $\kappa$ are given by^44^.

5 $$\begin{aligned} \gamma \equiv \frac{\mu _0\mu ^2\bar{\omega }}{2\hbar }Im[G_\perp (\textbf{r},\textbf{r};\bar{\omega })+G_ \parallel (\textbf{r},\textbf{r},\bar{\omega })]=\frac{1}{2}(\Gamma _\perp +\Gamma _ \parallel ), \end{aligned}$$

6 $$\begin{aligned} \kappa \equiv \frac{\mu _0\mu ^2\bar{\omega }}{2\hbar }Im[G_\perp (\textbf{r},\textbf{r};\bar{\omega })-G_ \parallel (\textbf{r},\textbf{r},\bar{\omega })]=\frac{1}{2}(\Gamma _\perp -\Gamma _ \parallel ), \end{aligned}$$

where $\small G_\perp =G_{zz}$ and $G_\parallel =G_{xx}$ denote the components of the electromagnetic Green’s tensor. The symbol $\small {\perp (\parallel )}$ refers to the orientation of a dipol that is normale along the Z-axis (parallel along the X-axis) to the surface of the nanostructure. In addition, $\textbf{r}$ is the position of the source, and $\mu _0$ denotes the permeability of the vacuum. We define the spontaneous emission rates as normal and parallel to the surface as $\small \Gamma _{\perp (\parallel )}=\mu _0\mu ^2\bar{\omega }Im [G_{\perp (\parallel )}(\textbf{r},\textbf{r};\bar{\omega })]\hbar$. The degree of quantum interference is determined by $V=(\Gamma _\perp -\Gamma _ \parallel )/(\Gamma _\perp +\Gamma _ \parallel )$. For $V=1$, the maximum quantum interference occurs in spontaneous emission, whereas by placing the emitter in vacuum $\Gamma _\perp =\Gamma _ \parallel$ and no quantum interference appears in the system^46^.

For the values of $\Gamma _{\perp }$ and $\Gamma _{\parallel }$ thar are used in this study, we refer to Fig. 3 in Ref.^27^. It is worth noting that the distance between the quantum system and the surface of the plasmonic nanostructure is a fraction of $c/\omega _{p}$, where $\omega _{p}$ and *c* are the bulk plasma frequency and speed of light, respectively. The reported results show that $\Gamma _{\perp }$ is larger than the free-space decay rate at the close distances to the plasmonic nanostructure, but as the distance increases (distances between $c/0.65\omega _{p}$ and $c/\omega _{p}$), the value of $\Gamma _{\perp }$ component decreases. Such a distinctive behavior is due to the Purcell effect.

The response of the system to the probe field is determined by the susceptibility which is defined as

7 $$\begin{aligned} \chi =\frac{2N\mu ^2}{\varepsilon _0\hbar }\frac{\rho _{20}}{\Omega _p}. \end{aligned}$$

where $\varepsilon _0$ and *N* are the vacuum permittivity and the atom number density in the medium, respectively. The parameter $\chi$ is complex, and its real and imaginary parts correspond to the dispersion and the absorption, respectively. The steady-state coherence term related to the probe field, $\rho _{20}$, can be derived via the analytical solution of the density matrix equations with respect to the weak amplitude fields as

8 $$\begin{aligned} \rho _{20}=-\frac{(2i(\gamma +\gamma ^{'}-i\delta )-\omega _{32})\Omega _p-2i\kappa \Omega _c}{4\kappa ^2-4(\gamma +\gamma ^{'}-i\delta )^2-\omega _{32}^2}. \end{aligned}$$

We substitute $\rho _{20}$ from Eq. (8) into Eq. (7) and obtain

9 $$\begin{aligned} \chi (\delta )=\frac{N\mu ^2}{\varepsilon _0\hbar } \left[ -\frac{2i(2(\gamma +\gamma ^{'}-i\delta )+i\omega _{32}-2\kappa \frac{\Omega _c}{\Omega _p})}{4\kappa ^2-4(\gamma +\gamma ^{'}-i\delta )^2-\omega _{32}^2}\right] . \end{aligned}$$

Now, we discuss the conditions for the exact optical transparency window. We first obtain the imaginary part of the susceptibility, Eq. (9) and then determine the conditions corresponding to zero absorption ($Im[\chi (\delta )]=0$) ,

10 $$\begin{aligned} Im[\chi (\delta )]=\frac{2N\mu ^2}{\varepsilon _0\hbar }\left( \frac{4(-4\nu \kappa ^2+4\kappa ^2\frac{\Omega _c}{\Omega _p}-\kappa \frac{\Omega _c}{\Omega _p}(\zeta -4\delta ^2+\omega _{32}^2)+\nu (\zeta +(-2\delta +\omega _{32})^2))}{64\nu ^2\delta ^2+(4\kappa ^2-4(\nu ^2-\delta ^2)-\omega _{32}^2)^2}\right) , \end{aligned}$$

where, $\nu =\gamma +\gamma ^{'}$ and $\zeta =4\gamma ^2+8\gamma \gamma ^{'}+4\gamma ^{'2}$. Equation (9) clearly shows how the plasmonic nanostructure affects the absorption and dispersion behavior of the probe field. An investigation of Eq. (10) reveals that the imaginary part of the susceptibility has basically nonzero magnitudes for the given parameters. However, amount of detuning leading to complete optical transparency, i.e. $Im[\chi (\delta )]=0$, is determined by

11 $$\begin{aligned} \delta =\frac{\nu \omega _{32}\pm \sqrt{-\zeta \nu ^2-4\kappa ^4(\frac{\Omega _c}{\Omega _p})^2+\kappa ^2(4\nu ^2+(\frac{\Omega _c}{\Omega _p})^2(\zeta +\omega _{32}^2))}}{2(\nu +\kappa (\frac{\Omega _c}{\Omega _p}))}. \end{aligned}$$

In fact, the Eq. (11) suggests the required values of detuning for zero absorption and non-zero dispersion in the presence of the plasmonic nanostructure.

Under the paraxial wave approximation, the propagation of probe beam is given by

12 $$\begin{aligned} \frac{\partial \Omega _p}{\partial {z}}=\frac{i}{2k_P}\left( \frac{\partial ^2}{\partial {x^2}}+\frac{\partial ^2}{\partial {y^2}}\right) \Omega _p+2i\pi {k_p}\chi \Omega _p. \end{aligned}$$

The first term in the right-hand of Eq. (12) refers to the diffraction, whereas the second term is responsible for the dispersion and absorption properties of the probe beam. We solve Eq. (12) numerically using a higher order split step operator method^47–49^ for probe beams whose initial shape is Gaussian with a width $W_p$ defined by $\Omega _p=\Omega _{p0}\exp {[-r^2/W_p^2]}$, where, $r=(\sqrt{x^2+y^2})$ is the transverse spatial coordinate. To study the behavior of the medium susceptibility as a function of the spatial variation of the control field intensity, we choose a doughnut-shaped LG control field as $\Omega _c=\Omega _{c0} \sqrt{r^2/W_c^2}\exp {[-r^2/W_c^2]}$, where $W_{c}$ is the beam waist. We assume that the intensity of the control beam is constant along the z-direction. We will propose different distances of the quantum system from the plasmonic nanostructure, where changing d leads to a change in the values of $\Gamma _\perp$ and $\Gamma _ \parallel$. The value of $\Gamma _\perp$ decreases as the distance between the quantum system and the plasmonic nanostructure increases. For distances closer to the plasmonic nanostructure, $\Gamma _\perp$ becomes much larger than the free-space decay rate. The values of parameters $\Gamma _\perp$ and $\Gamma _ \parallel$ for three different distances are given in Table 1.

**Table 1:** Values of $\Gamma _\perp$ and $\Gamma _ \parallel$ for three different distances between the quantum system and the surface of the plasmonic nanostructure.

Distance (nm)	$\Gamma _{\perp }/\Gamma _0$	${\Gamma _{\parallel }/\Gamma _0}$
31.2	1.774	0.021
41.6	0.559	0.021
52	0.196	0.026

## Results and discussion

We first present numerical results for the real (dispersion) and imaginary (absorption) parts of the susceptibility of the medium as a function of the probe field detuning. The aim is to show how the spatial structure of the control field in the presence of the plasmonic nanostructure modifies the propagation of the probe beam through the atomic system. It is important to note that the structure of the control field does not change significantly as it passes through the medium.

**Figure 2:**
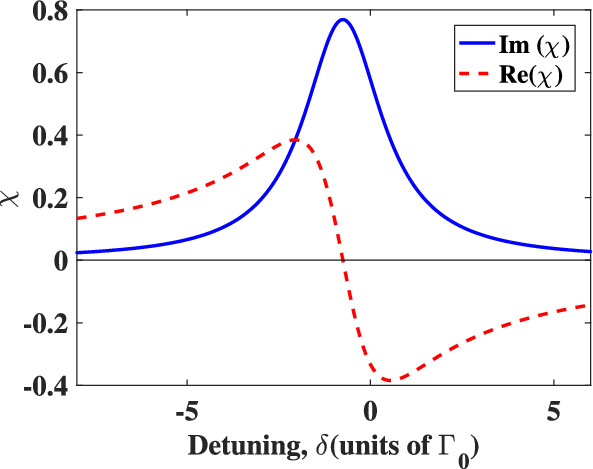
Absorption spectrum [$Im(\chi )$; solid curve] and dispersion spectrum [$Re(\chi )$; dashed curve] of the quantum system in units of $N\mu ^{'2}/(\varepsilon _0\hbar \Gamma _0)$, in the absence of the plasmonic nanostructure. We take $\omega _{32}=1.5\Gamma _0$ and $\gamma ^{'}=0.3\Gamma _0$.

Figure 2 shows the absorption and dispersion in the absence of the plasmonic nanostructure^44^. The parameters used in numerical calculations are $\Omega _{c0}/\Omega _{p0}=1.5$, $\omega _{32}=1.5\Gamma _0$, $\gamma ^{'}=0.3\Gamma _0$.

We note that there is no quantum interference in the system when the atomic system is in vacuum, corresponding to $\Gamma _\perp =\Gamma _\parallel$ and $\kappa =0$. Moreover, electromagnetically induced transparency is not created in the atomic system because the weak control field is applied to the atomic system. So, strong absorption of the probe field via the atoms is clear as can be seen in Fig. 2. However when the quantum system is in close proximity to the plasmonic nanostructure, the susceptibility of the medium behaves completely different.

**Figure 3:**
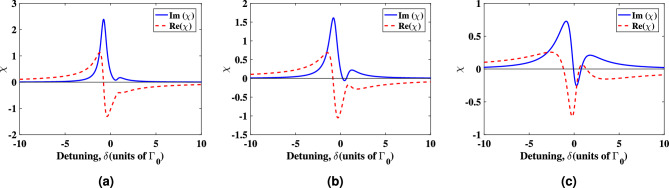
Absorption and dispersion spectra of the probe field in units of $N\mu ^{'2}/(\varepsilon _0\hbar \Gamma _0)$ in the presence of the plasmonic nanostructure at (**a**) $d=52$ nm, (**b**) $d=41.6$ nm (**c**) and $d=31.2$ nm, respectively. We take $\Omega _{c0}/\Omega _{p0}=1.5$, $\omega _{32}=1.5\Gamma _0$, $\gamma ^{'}=0.3\Gamma _0$ (**a**) $\Gamma _\perp =0.196\Gamma _0$ and $\Gamma _\parallel =0.026\Gamma _0$, (**b**) $\Gamma _\perp =0.559\Gamma _0$ and $\Gamma _\parallel =0.021\Gamma _0$, (**c**) $\Gamma _\perp =1.774\Gamma _0$ and $\Gamma _\parallel =0.021\Gamma _0$.

The absorption and dispersion spectra for various distances from the surface of the plasmonic nanostructure are shown in Fig. 3. Diagrams (a), (b) and (c) of Fig. 3 represent the results for the distances 52 nm, 41.6 nm and 31.2 nm between the quantum system and plasmonic nanostructure, respectively. The shape of the absorption and dispersion spectra is significantly influenced by the distance of the quantum system from the plasmonic nanostructure. As can be seen in Fig. 3a, the presence of the plasmonic nanostructure leads to reduced absorption and steepened dispersion near the line center. Optical transparency is achieved for d = 41.6 nm at the detunings $\delta =0.24, 1.29$ in agreement with the values obtained from Eq. (10). The shape of absorption and dispersion of the probe field in Fig. 3b,c differs from that seen in Fig. 3a because the values of $\Gamma _\perp$ and $\Gamma _\parallel$ change as the distance of plasmonic nanostructure from atomic system changes. At a distances of $R = 31.2$ nm and $R = 41.6$ nm, the imaginary part becomes negative, which leads to appearance of gain in the system. Figure 3 shows that the absorption diminishes and turns to the gain near the detuning line center as the atomic system approaches the plasmonic nanostructure. Furthermore, zero absorption and gain without inversion as well as nonzero dispersion, are attained in the cases of d = 41.6 nm and d = 31.2 nm. When an atom is placed close to plasmonic nanostructures, the quantum interference between two separate spontaneous emissions channels increases noticeably, and this is the main reason behind such behaviors.

**Figure 4:**
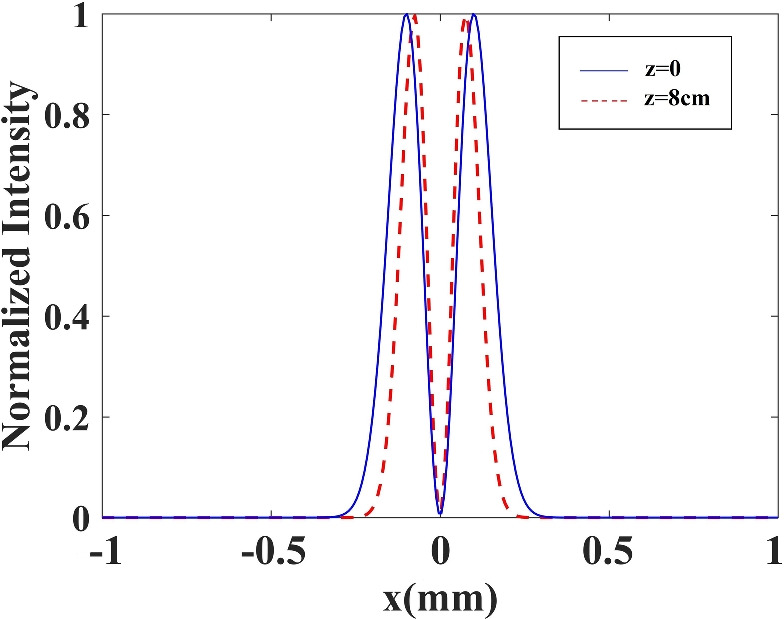
Peak normalized intensity profile of the input and propagated coupling beams. *r* is the radial distance from the beam center. $W_c=100\;\upmu$m, and the other parameters used are same as Fig. 2.

Figure 4 illustrates the variation of the normalized input and output intensities of the control field versus radial position. It is assumed that the control field passes through the atomic system with a length of 8 cm. Near the center, the control field has an intensity close to zero, while the maximum intensity values appear at position $|r|=0.01$ mm. Significant changes do not occur in the control field beam structure. So, the control field structure makes the atomic system inhomogeneous along the direction of the probe field propagation.

It was known that the spreading due to diffraction can be manipulated by propagating the light beam through a medium with spatially varying optical properties. Here, we consider a doughnut shaped LG control field to induce a waveguidelike refractive index pattern inside the atomic system.

**Figure 5:**
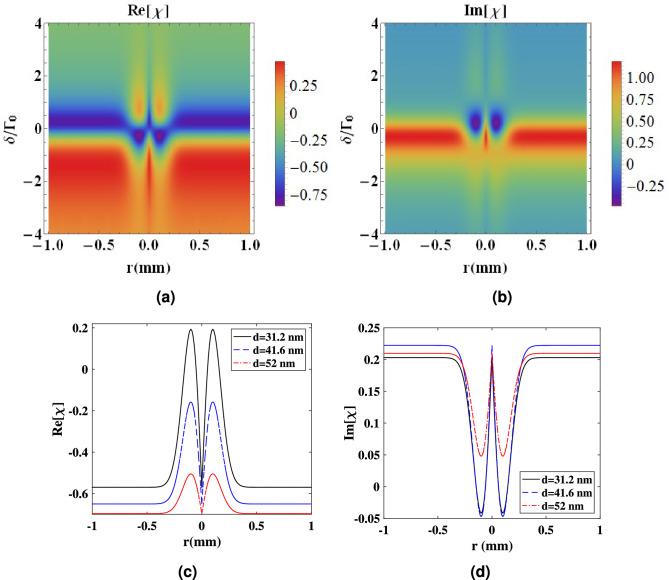
Three dimensional plot of (**a**) real and (**b**) imaginary parts of the susceptibility versus the probe field detuning and the radial distance from the beam center (*r*) in the presence of the plasmonic nanostructure at $d=31.2$ nm. (**c**) Dispersion and (**d**) absorption plots for distances $R = 31.2$ nm (solid curve), $R = 41.6$ nm (dashed curve) and $R = 52$ nm (dotted-dashed curve). The values used for the detuning of the probe field are $\delta = 0.57 \Gamma _{0}, 0.65 \Gamma _{0},$ and $0.79 \Gamma _{0}$ for distances $d=52$ nm, 41.6 nm and 31.2 nm, respectively.Other selected parameters are same as Fig. 3.

Figure 5 shows how the spatial structure of the control field allows us to guide the weak probe beam through the atomic system. It should be noted that no significant change occurs in the structure of the control field propagating through the medium. We analyze the spatial variation of the real and imaginary parts of the susceptibility using surface plots as a function of the radial position (*r*) and the probe field detuning for the atomic system placed at a distance of $d=31.2$ nm.

Figure 5a shows how changing the detuning can change the refractive index between negative and positive values. It is obvious from Fig. 5a that for the small positive detunings, the refractive index takes the high value at the diffraction ring and low values in the wings (radial sites around the center with large values of *r*). Note that where the intensity of the control field is large, a high refractive index occurs and the absorption undergoes a transition from high to low values as can be seen in Fig. 5b. Therefore, the LG control beam enables one to simulate a fiber-like refractive index profile inside the atomic system.

Figure 5c,d show the dispersion and absorption of the atomic system versus *r* for different values of distances between the plasmonic nanostructure and atomic system. We have used the proper values of detuning $\delta$ obtained from analytical solutions for each curve. The values used for the detuning of the probe field are $\delta = 0.57 \Gamma _{0}, 0.65 \Gamma _{0},$ and $0.79 \Gamma _{0}$ for distances $d=52$ nm, 41.6 nm and 31.2 nm, respectively.

From Fig. 5c, we find that the high value of the refractive index occurs in the region with the high control field intensity. The donut-shaped core is surrounded by the wing with a lower index of refraction. The value of the refractive index decreases with the reduction of the control field at the wings ($|r|>0.2$ mm) that leads to a waveguide-like behavior. Moreover, it can be seen from Fig. 5d that the absorption of the medium has a minimum at the region with high control field intensity. We find that an increase or decrease from the selected detuning results in increased absorption by the medium. The absorption becomes a gain at radial positions around the center for close distances between the plasmonic nanostructure and atomic system.

By changing the distance of the plasmonic nanostructure from the atomic system, the quantum interference between two spontaneous emission channels of three-level V-type atomic system changes and subsequently influences the response of the atoms to the probe light propagation.

It is worth noting that the refractive index sharply varied by placing the plasmonic nanostructure at closer distances to the atomic system. So, the plasmonic nanostructure strongly affects the optical properties of the atomic system and generates a high-contrast waveguide with the help of the spatial variation of the susceptibility.

**Figure 6:**
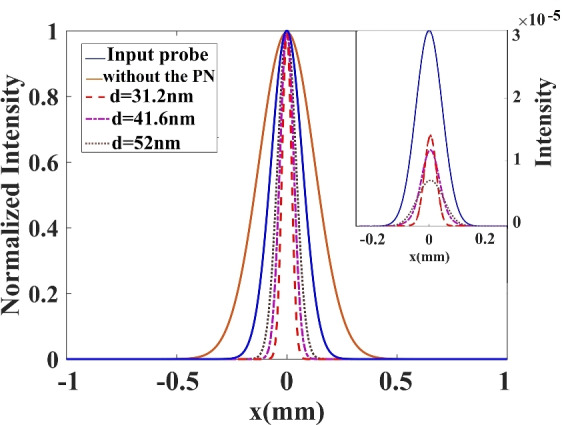
Peak normalized intensity profile of the propagated probe beam (output beam) in the presence and absence of the plasmonic nanostructure (PN). The blue solid curve shows the input intensity, the brown solid curve corresponds to the case without the plasmonic nanostructure, the dotted curve corresponds to distance $R = 52$ nm, the dotted-dashed curve corresponds to $d=41.6$ nm, and the dashed curve corresponds to $d=31.2$ nm. $W_p=W_c=100\;\upmu$m, and the other parameters used are the same as Fig. 3. The values used for the detuning of the probe field are $\delta = 0.57 \Gamma _{0}, 0.65 \Gamma _{0},$ and $0.79 \Gamma _{0}$ for distances $d=52$ nm, 41.6 nm and 31.2 nm, respectively. The inset shows the variation of the unnormalized input and output intensities of the probe beam.

Now, by using a higher-order split operator approach to solve the paraxial wave Eq. (12), we investigate the influence of inhomogeneous susceptibility on the probe beam propagation. At the entrance surface of the vapor cell, the widths of the control and probe beams are assumed to be the same, $W_p=W_c=100\;\upmu$m. The normalized intensity of the transmitted probe beam is plotted versus x for y = 0 and different distances d in Fig. 6. The profile at the input of the vapor cell is shown by the thick solid line. The output profile after 8 cm propagation length in the absence of the plasmonic nanostructure is depicted by the solid line. The dashed line is displayed for d = 31.2 nm, while the dash-dotted curve and dotted curve are obtained for the plasmonic nanostructure placed at d = 41.6 nm and d = 52 nm from the atomic system, respectively. Using Eq. (10), we can determine the exact detunings at which the probe field is effectively transmitted without absorption in the presence of the plasmonic nanostructure.

The FWHM of the probe beam, which is corresponds to 0.96 mm in the presence of the plasmonic nanostructure, indicates a 45% reduction in the probe beam due to beam focusing. Additionally, the FWHM of the probe beam narrows and sees a 70% drop in the presence of the plasmonic nanostructure at closer distances from the atomic system.

We also compare the output intensity of the probe beam after traversing a propagation length of 8 cm for various distances from the surface of the plasmonic nanostructure in the presence of a LG control field. Variation of the unnormalized output intensity of the probe beam is shown in the inset of Fig. 6. It can be seen that by placing the plasmonic nanostructure at a distance of 32 nm from the atomic system, the diffraction-less beam could pass through the medium with 50% transmittance. As the distance between the nanostructure and the atomic system increases, the amount of light transmission decreases. These results are completely in agreement with the results obtained in Fig. 5. As can be seen from Fig. 5, the minimum absorption for the peak of the probe beam occurs at the closest distance of the atoms from the plasma plane. The reason for this behavior is that the quantum interference is stronger at a closer distance and has a significant impact on the properties of the quantum system and the light passing through it.

**Figure 7:**
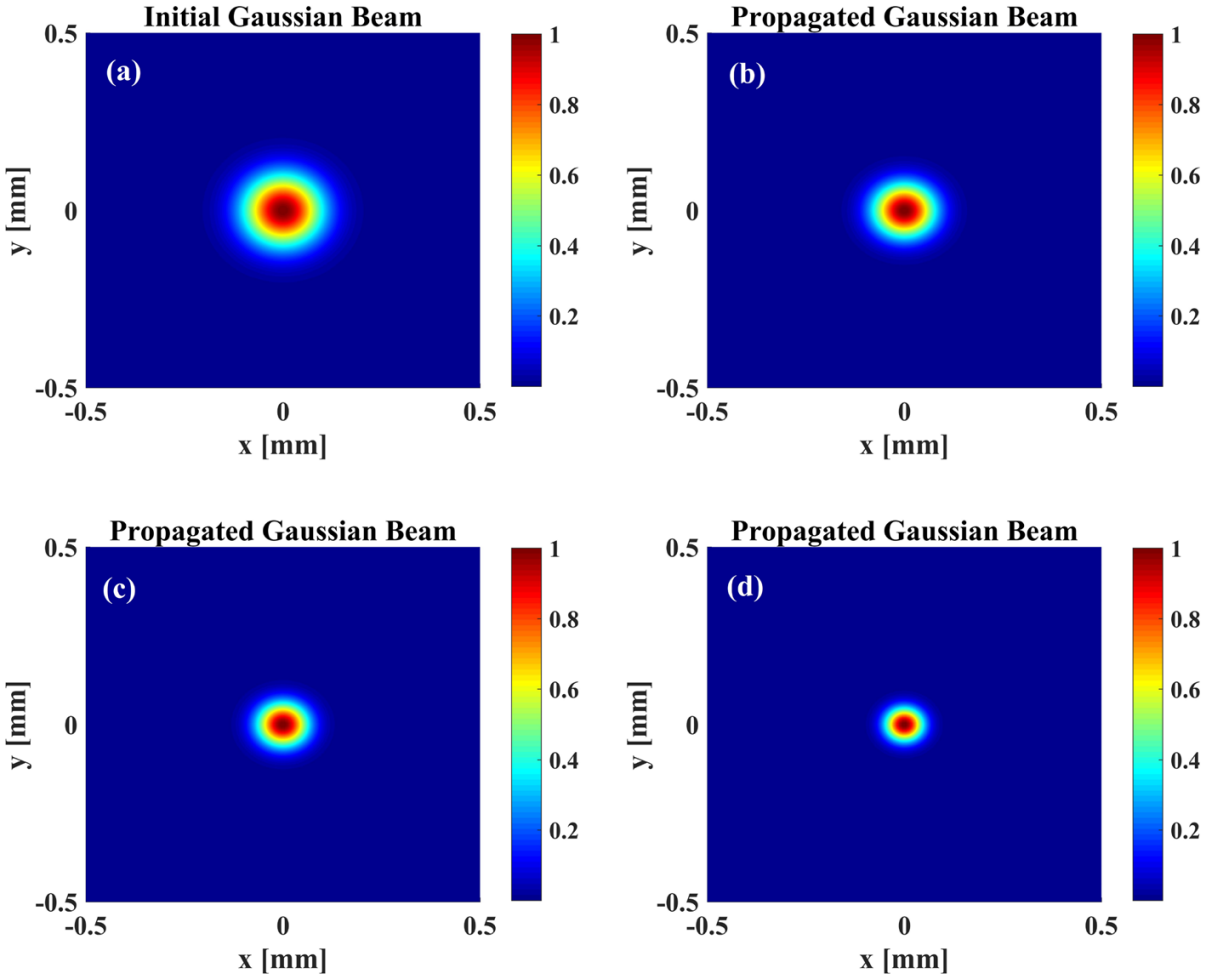
(**a**) Input and output propagated probe beam for (**b**) $d=52$ nm, (**c**) $d=41.6$ nm, and (**d**) $d=31.2$ nm. The other parameters used are same as Fig. 6.

Figure 7 demonstrates the 3D initial and output probe beams that are illustrated in Fig. 6. Figure 7 represents the radial fluctuation of probe beam intensity in the presence of the plasmonic nanostructures. Figure 7a–d show the initial Gaussian probe beam and the propagated Gaussian probe beams at $d=52$ nm, $d=41.6$ nm, and $d=31.2$ nm, respectively. We find that as the plasmonic nanostructure approaches the atomic system, the amount of light focusing increases. It can be seen that applying the LG control beam in the presence of the plasmonic nanostructure results in maintaining the shape of the passing Gaussian beam.

The obtained results show that with the help of two factors, the control field with spatial structure and the plasmonic nanostructure, the propagation of a diffraction-limited light through the atomic system can be observed.

## Conclusion

We study the diffractionless propagation of a Gaussian beam through a four-level atomic system placed near a plasmonic nanostructure. Spatially modulated susceptibility that is created by the LG control beam causes adjusted dispersion experienced by the probe beam. We demonstrate that the presence of the plasmonic nanostructure results in a significant manipulation of the absorption and dispersion spectra of the probe field. This behavior is achieved by ability of the plasmonic nanostructure to generate quantum interference between channels of spontaneous emission. Furthermore, simply changing the distance between the atomic system and the plasmonic nanostructure in the quantum system generates zero absorption with nonzero dispersion and amplification without inversion. We have shown that, in the presence of the plasmonic nanostructure, the application of a control beam with LG profile results in the diffraction-limited probe light propagating.

## Data Availability

The datasets used and analysed during the current study are available from the corresponding author on reasonable request.

## References

[1] Dey T, Agarwal G (2009). Subdiffraction propagation of images using saturated absorption of optical transition. Opt. Lett..

[2] Zhang L, Dey TN, Evers J (2013). Control of beam propagation in optically written waveguides beyond the paraxial approximation. Phys. Rev. A.

[3] Firstenberg O, Shuker M, Davidson N, Ron A (2009). Elimination of the diffraction of arbitrary images imprinted on slow light. Phys. Rev. Lett..

[4] Cheng J, Han S (2007). Electromagnetically induced self-imaging. Opt. Lett..

[5] Kapoor R, Agarwal G (2000). Theory of electromagnetically induced waveguides. Phys. Rev. A.

[6] Li H (2008). Optical imaging beyond the diffraction limit via dark states. Phys. Rev. A.

[7] Ding D-S, Zhou Z-Y, Shi B-S (2014). Image cloning beyond diffraction based on coherent population trapping in a hot rubidium vapor. Opt. Lett..

[8] Kapale KT, Agarwal GS (2010). Subnanoscale resolution for microscopy via coherent population trapping. Opt. Lett..

[9] Kiffner M, Evers J, Zubairy M (2008). Resonant interferometric lithography beyond the diffraction limit. Phys. Rev. Lett..

[10] Nilsson M, Kröll S (2005). Solid state quantum memory using complete absorption and re-emission of photons by tailored and externally controlled inhomogeneous absorption profiles. Opt. Commun..

[11] Vudyasetu PK, Starling DJ, Howell JC (2009). All optical waveguiding in a coherent atomic rubidium vapor. Phys. Rev. Lett..

[12] Truscott A, Friese M, Heckenberg N, Rubinsztein-Dunlop H (1999). Optically written waveguide in an atomic vapor. Phys. Rev. Lett..

[13] Andersen J (2001). Light guiding light: Nonlinear refraction in rubidium vapor. Phys. Rev. A.

[14] Apolinario UF, Manzoor S, de Araujo LE (2017). Optical image cloning based on electromagnetic induced absorption. Opt. Lett..

[15] Vengalattore M, Prentiss M (2005). Radial confinement of light in an ultracold anisotropic medium. Phys. Rev. Lett..

[16] Verma, O. N. & Roy, S. Microwave field controlled electromagnetically induced focusing. *Japan. J. Appl. Phys*. **57**, 08PF01 (2018).

[17] Dey TN, Evers J (2011). Nondiffracting optical beams in a three-level Raman system. Phys. Rev. A.

[18] Sharma S (2023). Efficient diffraction control using a tunable active-Raman gain medium. Phys. Rev. A.

[19] Sharma S, Dey TN (2017). Kerr-field-induced tunable optical atomic waveguide. Phys. Rev. A.

[20] Vafafard A, Sahrai M, Asadpour SH, Faizabadi E (2021). Tunable magneto-optical faraday rotation with a five-level atomic system near the plasmonic nanostructure. JOSA B.

[21] Chang D, Sørensen AS, Hemmer P, Lukin M (2006). Quantum optics with surface plasmons. Phys. Rev. Lett..

[22] Trügler A, Hohenester U (2008). Strong coupling between a metallic nanoparticle and a single molecule. Phys. Rev. B.

[23] Gonzalez-Tudela A, Rodriguez FJ, Quiroga L, Tejedor C (2010). Dissipative dynamics of a solid-state qubit coupled to surface plasmons: From non-markov to markov regimes. Phys. Rev. B.

[24] Esteban, R., Laroche, M. & Greffet, J.-J. Influence of metallic nanoparticles on upconversion processes. *J. Appl. Phys*. **105** (2009).

[25] Yannopapas V (2010). Enhancement of nonlinear susceptibilities near plasmonic metamaterials. Opt. Commun..

[26] Gersten JI, Nitzan A (1985). Photophysics and photochemistry near surfaces and small particles. Surface Sci..

[27] Evangelou S, Yannopapas V, Paspalakis E (2012). Transparency and slow light in a four-level quantum system near a plasmonic nanostructure. Phys. Rev. A.

[28] Agarwal, G. & ONeil, S. Effect of hydrodynamic dispersion of the metal on surface plasmons and surface-enhanced phenomena in spherical geometries. *Phys. Rev. B*. **28**, 487 (1983).

[29] Anger P, Bharadwaj P, Novotny L (2006). Enhancement and quenching of single-molecule fluorescence. Phys. Rev. Lett..

[30] Fedutik Y, Temnov V, Schöps O, Woggon U, Artemyev M (2007). Exciton-plasmon-photon conversion in plasmonic nanostructures. Phys. Rev. Lett..

[31] Kühn S, Mori G, Agio M, Sandoghdar V (2008). Modification of single molecule fluorescence close to a nanostructure: Radiation pattern, spontaneous emission and quenching. Mol. Phys..

[32] Vasa P (2008). Coherent exciton-surface-plasmon-polariton interaction in hybrid metal-semiconductor nanostructures. Phys. Rev. Lett..

[33] Ringler M (2008). Shaping emission spectra of fluorescent molecules with single plasmonic nanoresonators. Phys. Rev. Lett..

[34] Wang Y, Yang T, Tuominen MT, Achermann M (2009). Radiative rate enhancements in ensembles of hybrid metal-semiconductor nanostructures. Phys. Rev. Lett..

[35] Hakala T (2009). Vacuum rabi splitting and strong-coupling dynamics for surface-plasmon polaritons and rhodamine 6g molecules. Phys. Rev. Lett..

[36] Gomez D, Vernon K, Mulvaney P, Davis T (2010). surface plasmon mediated strong exciton–photon coupling in semiconductor nanocrystals. Nano Lett..

[37] Hatef A, Singh MR (2010). Plasmonic effect on quantum coherence and interference in metallic photonic crystals doped with quantum dots. Phys. Rev. A.

[38] Sadeghi S (2009). The inhibition of optical excitations and enhancement of rabi flopping in hybrid quantum dot-metallic nanoparticle systems. Nanotechnology.

[39] Thanopulos I, Paspalakis E, Yannopapas V (2012). Plasmon-induced enhancement of nonlinear optical rectification in organic materials. Phys. Rev. B.

[40] Evangelou S, Yannopapas V, Paspalakis E (2011). Modifying free-space spontaneous emission near a plasmonic nanostructure. Phys. Rev. A.

[41] Vafafard A, Sahrai M, Siahpoush V, Hamedi HR, Asadpour SH (2020). Optically induced diffraction gratings based on periodic modulation of linear and nonlinear effects for atom-light coupling quantum systems near plasmonic nanostructures. Sci. Rep..

[42] Wang H, Kundu J, Halas NJ (2007). Plasmonic nanoshell arrays combine surface-enhanced vibrational spectroscopies on a single substrate. Angewandte Chemie Int. Edn..

[43] Yang S, Cai W, Kong L, Lei Y (2010). Surface nanometer-scale patterning in realizing large-scale ordered arrays of metallic nanoshells with well-defined structures and controllable properties. Adv. Funct. Mater..

[44] Paspalakis E, Evangelou S, Yannopapas V, Terzis AF (2013). Phase-dependent optical effects in a four-level quantum system near a plasmonic nanostructure. Phys. Rev. A.

[45] Lee H, Polynkin P, Scully MO, Zhu S-Y (1997). Quenching of spontaneous emission via quantum interference. Phys. Rev. A.

[46] Kiffner, M., Macovei, M., Evers, J. & Keitel, C. Vacuum-induced processes in multilevel atoms. In *Progress in Optics*, vol. 55, 85–197 (Elsevier, 2010).

[47] Bandrauk A, Shen H (1994). High-order split-step exponential methods for solving coupled nonlinear schrodinger equations. J. Phys. A Math. General.

[48] Balac S, Mahé F (2015). An embedded split-step method for solving the nonlinear schrödinger equation in optics. J. Comput. Phys..

[49] Weideman J, Herbst BM (1986). Split-step methods for the solution of the nonlinear schrödinger equation. SIAM J. Numer. Anal..

